# Ocular Syphilis in Individuals with and Without HIV: A Single-Center University Hospital Experience

**DOI:** 10.3390/pathogens15030256

**Published:** 2026-02-27

**Authors:** Murat Hakan Kır, Aysun Benli, Zarifa Orta, Seniha Başaran, Merih Oray, Halit Özsüt

**Affiliations:** 1 Department of Infectious Diseases and Clinical Microbiology, Istanbul Faculty of Medicine, Istanbul University, Istanbul 34320, Türkiye; aysun.benli@istanbul.edu.tr (A.B.);; 2 Department of Infectious Diseases and Clinical Microbiology, Tekirdag Dr. Ismail Fehmi Cumalioglu City Hospital, Tekirdag 59030, Türkiye; 3 Department of Ophthalmology, Faculty of Medicine, Yeni Yuzyil University, Istanbul 34010, Türkiye

**Keywords:** ocular syphilis, HIV, uveitis, neurosyphilis, visual loss

## Abstract

Syphilis is a re-emerging sexually transmitted infection with rising incidence worldwide, often associated with HIV infection. Ocular syphilis represents a severe manifestation that can occur at any disease stage and may result in permanent vision loss if not promptly diagnosed and treated. We conducted a retrospective comparative cohort study of 22 patients with ocular syphilis managed at Istanbul University, Istanbul Faculty of Medicine, between 2019 and 2025. Twelve patients (54.5%) were people living with HIV (PLWH). The majority were male (81.8%), with a mean age of 45.2 years. Visual loss was observed in more than half of the patients and occurred significantly more frequently in PLWH than in HIV-negative individuals (100% vs. 70%; *p* = 0.046). Vitritis was also significantly more frequent among PLWH (91.7% vs. 40%; *p* = 0.02), indicating more severe intraocular inflammation. All six cases of neurosyphilis were confined to PLWH (50% vs. 0%; *p* = 0.004). The most common ocular manifestations were uveitis (90.9%), predominantly panuveitis and posterior uveitis. All patients received intravenous penicillin G or ceftriaxone, and systemic corticosteroids were administered in half of the cases. Clinical improvement was observed in all patients. Our findings highlight that ocular syphilis in PLWH is associated with more severe inflammation and higher neurosyphilis risk, underscoring the importance of routine cerebrospinal fluid examination and neurosyphilis-based treatment strategies in this group.

## 1. Introduction

Syphilis is a disease caused by *T. pallidum*, transmitted primarily through sexual contact and direct contact, and rarely through vertical (fetal) transmission and blood transfusion [1]. Syphilis, historically documented since the late 15th century, has re-emerged as a major global public health concern over the past decade. According to the World Health Organization (WHO), approximately 8 million new syphilis infections occurred worldwide in 2022, reflecting a sustained global resurgence despite the availability of effective prevention and treatment strategies [2]. Parallel to global trends, Turkey has also experienced a marked increase in syphilis cases. National surveillance data from the Turkish Ministry of Health indicate that reported syphilis cases rose more than tenfold, from 281 cases in 2012 to 3646 cases in 2023, highlighting a substantial and ongoing epidemiological shift [3]. The increase in syphilis incidence, along with the rise in HIV infection in recent years, is concerning. In particular, with the introduction of effective antiretroviral therapy (ART) regimens, AIDS-related mortality rates have declined, and the frequency of unprotected sex among men who have sex with men (MSM) with HIV RNA-negative status has increased. These two key factors are thought to be responsible for the increase in syphilis incidence [4]. According to WHO data, the prevalence of syphilis among MSM in countries reporting syphilis in 2019 was found to be 11.8% on average. According to European data, MSM individuals account for 77% of syphilis cases, a significant increase from 62% in 2015. The increase in the use of pre-exposure prophylaxis (PrEP) among MSM individuals [5] may have led to an increase in syphilis infection [6]. This increase may be attributed to an increase in the frequency of syphilis testing among individuals using PrEP, leading to an increase in the frequency of diagnosis, and an increase in risky sexual behavior among individuals using PrEP. It is known that HIV infection, which is frequently associated with syphilis, is also on the rise in Turkey. Syphilis, which can present with a wide variety of clinical manifestations, is primarily classified into primary, secondary, latent, and tertiary stages. Neurosyphilis, which can be seen in every stage of syphilis, is defined as the involvement of the central nervous system (CNS) by *T. pallidum* [7]. Syphilis can also manifest itself with eye involvement, which is not uncommon, and ocular syphilis, like neurosyphilis, can occur in every stage of syphilis. Acute ocular inflammation is more common in the primary, secondary, and early latent stages of syphilis, while chronic ocular inflammation is seen in the late latent and tertiary stages of syphilis, defined as late syphilis [8]. Ocular syphilis was previously considered a subtype of neurosyphilis, but it is now considered a separate entity and can occur independently of neurosyphilis [9]. Although it can affect any layer of the eye, it most commonly presents as posterior uveitis and panuveitis [10]. A report published in the US in 2015 indicated an increase in ocular syphilis [11]. Since ocular syphilis is a serious infectious disease that can lead to vision loss, rapid diagnosis and treatment are critical, and awareness of the disease among patients and physicians should be increased. In our faculty, based on the observational increase in ocular syphilis cases in recent years, the aim was to evaluate the characteristics of People Living with HIV(PLWH) and HIV-negative ocular syphilis cases who were treated and followed up.

## 2. Materials and Methods

This study was designed as a retrospective comparative cohort study. The epidemiological, clinical, and laboratory data of ocular syphilis cases monitored by the Department of Infectious Diseases and Clinical Microbiology between 2019 and 2025 were retrospectively collected using standardized data collection forms from inpatient medical records and the hospital automation system. The diagnosis of ocular syphilis was established based on positive serological tests (VDRL, TPHA, FTA-ABS) and findings consistent with ocular syphilis on eye examinations. In all patients, non-treponemal (VDRL) and treponemal (TPHA and FTA-ABS) serological tests were performed. The diagnosis of ocular syphilis was established based on compatible ocular clinical findings together with reactive treponemal serology; concordant positivity of all serological assays was not required for case definition. All cases were evaluated jointly by infectious disease specialists and ophthalmologists. The diagnosis of ocular syphilis was established based on compatible clinical findings, reactive treponemal serology, in accordance with CDC guideline recommendations and after exclusion of alternative infectious causes [12,13].

The diagnosis of neurosyphilis was made based on the detection of more than 20 cells/mm^3^ in the cerebrospinal fluid (CSF) in people living with HIV (PLWH), more than 5 cells/mm^3^ in HIV-negative individuals, or a positive CSF serological test (VDRL or FTA-ABS) [14,15]. CSF evaluation included cell count and serological testing in patients who underwent lumbar puncture, and fulfillment of any one of the predefined criteria was considered sufficient for the diagnosis of neurosyphilis; concordant positivity of CSF pleocytosis and serological tests was not required. In patients presenting with neurological or severe ocular manifestations, additional investigations were performed to exclude alternative infectious etiologies, including *Cryptococcus neoformans*, *Toxoplasma gondii*, HSV, and VZV, using cerebrospinal fluid analysis, serological tests, and neuroimaging when clinically indicated. Patient data including sex, age, duration of symptoms, HIV status, presence of visual complaints, unilateral or bilateral eye involvement, ocular structure involvement, vision loss on examination, stage of syphilis, concomitant neurosyphilis status, performance of lumbar puncture, laboratory parameters, and antibiotic and corticosteroid treatment were recorded. Individuals under 18 years of age were excluded from the study. Comparisons between PLWH and HIV-negative patients with ocular syphilis were performed using appropriate statistical tests. Statistical analyses were conducted using Statistical Package for the Social Sciences (SPSS) version 26 (IBM Corp., Armonk, NY, USA). Categorical variables were analyzed using the chi-square or Fisher’s exact test, while continuous variables were compared using the Mann–Whitney U test. Where appropriate, odds ratios (OR) with 95% confidence intervals (CI) were calculated, and a two-sided *p* value < 0.05 was considered statistically significant.

## 3. Results

A total of 22 cases of ocular syphilis were observed during the specified study period. The characteristics of the patients in the study are listed in Table 1 and the sites of eye involvement are in Table 2. Comparative analyses between PLWH and HIV-negative individuals revealed statistically significant differences in several key clinical and laboratory parameters.

Methylprednisolone and prednisolone were used as corticosteroid therapy in oral or IV forms, with prednisolone doses ranging from 15 to 40 mg and methylprednisolone doses ranging from 8 to 40 mg. None of the HIV-negative patients were using PrEP. Vision loss was significantly more common in individuals living with HIV than in HIV-negative individuals (100% vs. 70%; *p* = 0.046; OR: 5.1; CI: 1.0–27.2). Vitritis was more common in PLWH than in HIV-negative individuals (91.7% vs. 40.0%; *p* = 0.02; OR: 7.0; CI: 1.1–45.2), indicating more severe ocular inflammation in PLWH. Four patients had neurological complaints/findings accompanying ocular findings. Two patients had headaches, two had dizziness/imbalance, one had memory loss, and one had diplopia, dysphagia, and peripheral facial paralysis. Six patients were diagnosed with neurosyphilis, all of whom were PLWH (50% vs. 0%; *p* = 0.004). Compared with HIV-negative individuals, PLWH presented earlier after symptom onset and showed significantly higher rates of vitritis, vision loss, neurosyphilis, and positive CSF serological findings. Among patients who underwent lumbar puncture, cerebrospinal fluid FTA-ABS positivity was detected in 5 of 10 patients (50%), all of whom were PLWH, while none of the HIV-negative patients showed FTA-ABS positivity (*p* = 0.010). Similarly, CSF VDRL positivity was observed in 3 of 11 patients (27.3%), all in the PLWH group, with no positive cases among HIV-negative individuals (*p* = 0.020). Only one of the 22 patients had involvement of the seventh cranial nerve. All PLWH patients had CD4+ T lymphocyte counts above 200. Seven of the 12 PLWH were receiving ART when they were diagnosed with ocular syphilis, while the other five patients were diagnosed with HIV at the same time as ocular syphilis. All patients who had previously started ART had been screened for syphilis, and none of them had previously been diagnosed with syphilis. Initial blood VDRL titers ranged from 1/32 to 1/128. Crystallized penicillin G 6 × 4 million U IV was used in the treatment of all patients except one; one patient received ceftriaxone 2 × 2 g IV treatment due to allergy. The total duration of treatment ranged from 14 to 21 days. Complete or partial improvement in ocular findings was observed in all treated patients.

## 4. Discussion

In this study, we compiled cases of ocular syphilis, which has been on the rise in recent years but has low awareness among physicians and the general public. We sought to examine differences in epidemiological, clinical, and physical examination findings between HIV-positive and HIV-negative individuals with ocular syphilis cases observed at our center, whether neurosyphilis accompanied ocular syphilis, and the treatment characteristics of these patients. The time from symptom onset to hospital presentation was significantly shorter in PLWH compared to HIV-negative patients. In addition to increased disease severity, this shorter time to presentation may also reflect greater health awareness and more frequent engagement with healthcare services among PLWH due to regular follow-up visits. This could be explained by better awareness of syphilis in PLWH, their regular hospital visits, or the presentation being faster due to more severe inflammation findings associated with ocular syphilis. The proportion of PLWH among our ocular syphilis cases is comparable to reports from Europe and North America, where HIV co-infection rates range from 40% to 60% [10,11]. Notably, our findings also align with data from other middle-income countries, suggesting that HIV-associated ocular syphilis represents a global clinical challenge. These results underscore the relevance of systematic HIV screening and integrated management strategies across different healthcare settings, including Türkiye. There is no epidemiological data available on ocular syphilis cases in Turkey. Only two case series have been reported in Turkey previously, and our series is the largest to date. Given the scarcity of data in the country, it is believed that this study will contribute to the Turkish data. Unlike the data from the ocular syphilis study group, the most common stage of syphilis presentation in our study was latent syphilis, not secondary syphilis [16]. Although it is difficult to reach a general conclusion with such a small number of patients, the association between HIV coinfection and ocular syphilis was not statistically significant. While some studies report ocular syphilis being more common in HIV-positive individuals [11] other studies did not observe such a pattern [17]. In our study, as in other studies, panuveitis and posterior uveitis were the most common findings [18,19]. Contrary to most studies, no significant correlation was observed in our study between anterior uveitis and HIV infection [20,21]. Consistent with other studies in the literature, ocular inflammation was more common in PLWH in our study a finding that supports the current literature [22]. This finding may be explained by persistent immune dysregulation, chronic inflammation, and altered cellular immunity in PLWH, even in the presence of effective antiretroviral therapy. These factors may facilitate deeper tissue invasion by *Treponema pallidum* and a more pronounced inflammatory response within ocular structures. In our study, no significant correlation was observed between bilateral involvement and HIV status. While some publications show a correlation between bilateral involvement and HIV seropositivity [23,24], some studies do not [17,22]. It is clear that more studies are needed regarding the relationship between bilateral involvement and HIV seropositivity. In our study, the rate of vision loss detected during examination was significantly higher in PLWH than in HIV-negative individuals; this may be due to more severe ocular inflammation in PLWH. More severe intraocular inflammation, delayed diagnosis in some cases, and possible microvascular involvement may collectively contribute to the higher risk of visual impairment in this population. The higher incidence of vitritis in PLWH also supports this finding. Considering that 5 patients in our study were diagnosed with HIV concurrently with ocular syphilis, clinicians should bear in mind that ocular syphilis may be the first manifestation in HIV patients. The higher incidence of neurosyphilis in PLWH supports the recommendation that LP should be considered in patients with ocular syphilis. All PLWH in our cohort had CD4+ T-cell counts above 200 cells/mm^3^, suggesting preserved immune function. Since we have no patients who have previously been diagnosed with syphilis, we have no patients in whom we suspect worsening of IRIS-associated ocular syphilis findings. HIV-related disruption of the blood–brain barrier and impaired central nervous system immune surveillance may increase susceptibility to central nervous system involvement by *T. pallidum*, thereby contributing to the higher frequency of neurosyphilis observed in PLWH. Neurosyphilis may be more common in individuals living with HIV due to immunosuppression [25,26]. In our study, while there was no difference in the number of ocular syphilis cases between HIV-infected and non-infected individuals, all cases of neurosyphilis accompanying ocular syphilis were in PLWH. As in our study, some studies have also reported that neurosyphilis is more common in PLWH [10,27]. However, in some studies inconsistent with our study, neurosyphilis was not more common in PLWH diagnosed with ocular syphilis [13]. Therefore, it is clear that more studies are needed to reach a definitive conclusion and that more precise diagnostic criteria for neurosyphilis need to be established for PLWH and HIV-negative patients. The CDC recommends that LP be performed in all cases of ocular syphilis, regardless of neurological findings, and that treatment be organized as for neurosyphilis, regardless of the presence or absence of pleocytosis [12]. However, the necessity of performing CSF examination in all cases of ocular syphilis remains controversial. The European Syphilis Treatment Guidelines recommend that LP be performed in all cases of PLWH diagnosed with ocular syphilis [13]. Despite this recommendation, the rate of routine LP in ocular syphilis cases in the ocular syphilis study group was reported as 40.8%, while in our study, this rate was 72.7%. This may be because the rate of consultation with an infectious disease specialist in the ocular syphilis working group was 62%, while in our study, this rate was 100%. This finding demonstrates the importance of infectious disease consultation in the management of ocular syphilis. In our patient group, treatment durations ranged from 14 to 21 days, and cases were treated in accordance with the recommendations. In our study, IV penicillin G was administered to all patients except one, and IM procaine penicillin treatment was not used. In the ocular syphilis study group, the rate of IM procaine penicillin use was reported as 25% [16]. This may be related to countries’ insurance systems, hospitalization difficulties, and clinician experience. The 3 doses of IM procaine penicillin recommended by the NHS can be considered as an alternative approach when patients cannot be hospitalized. None of the HIV-negative patients were using PrEP, which may indicate that the use of PrEP is still not widespread in Turkey and that awareness of PrEP is low [28]. Among the reasons for the low use of PrEP in Turkey are the lack of insurance reimbursement, high drug prices due to the current exchange rate, and Turkey not being an open-minded country regarding LGBT rights [29]. In particular, MSM individuals’ lack of knowledge about PrEP and their fear of stigmatization can be cited as reasons for this situation [30]. The lack of sufficient data on PrEP use in Turkey also highlights the need for further research on this topic. Despite the limited sample size, the comparative nature of our analysis provides clinically meaningful insights into HIV-associated differences in ocular syphilis severity.

## 5. Limitations

The main limitation of our study is that it is a single-center, retrospective study; specifically, the limited number of patients. The Introduction Section states that syphilis is on the rise, particularly among MSM infected with HIV. Unfortunately, since the patients’ sexual orientation history was missing in our study, it was not possible to examine this aspect. The improvement processes in the eye findings during the patients’ outpatient follow-ups could not be included in the study due to irregularities in patient visits to, requests for follow-up at other centers, and data deficiencies. In addition, standardized long-term follow-up data were not uniformly available due to the retrospective design and incomplete outpatient records, which limited detailed assessment of long-term clinical outcomes. Due to the absence of PLWH with CD4+ T lymphocyte counts below 200 mm^3^, the differences in ocular syphilis characteristics between patients in the AIDS stage and those who were not could not be examined. The correlation between HIV RNA levels and disease severity could not be meaningfully examined due to the small number of patients.

## 6. Conclusions

In conclusion, ocular syphilis is an infectious disease with low awareness among both the general public and physicians, and its incidence is increasing. Considering that HIV incidence is also increasing in Turkey, an increase in ocular syphilis cases can be anticipated. It is essential to prevent these cases, ensure accurate and early diagnosis, and provide appropriate treatment. As seen in our study, ocular syphilis in PLWH individuals is associated with more severe ocular inflammation and a higher rate of neurosyphilis. Therefore, when ocular syphilis is detected, especially in PLWH individuals, cerebrospinal fluid (CSF) examination should be strongly considered, and treatment should be organized according to neurosyphilis protocols. Furthermore, syphilis screening when visual complaints develop in PLWH is critically important for prognosis.

## Figures and Tables

**Table 1:** Characteristics of HIV-positive and HIV-negative individuals diagnosed with ocular syphilis.

	All Patients (*n* = 22)	PLWH (12)	HIV Negative (10)	*p*
Average Age (*n*)	45.18	39.33	52.2	0.245
Male Gender *n* (%)	18 (82)	11 (91.7)	7 (70)	0.200
Time to Presentation After Symptom Onset (days)	68	24	341	**0.010**
Visual Impairment Complaint, *n* (%)	12 (54.5)	7 (58.3)	5 (50)	0.703
Bilateral Eye Involvement, *n* (%)	13 (59.0)	8 (66.7)	5 (50)	0.408
Eye Involvement				
- Chorioretinitis, *n* (%)	12 (54.5)	8 (66.7)	4 (40)	0.258
- Uveitis, *n* (%)	20 (90.9)	11 (91.7)	9 (90)	0.895
- Vitritis, *n* (%)	15 (68.2)	11 (91.7)	4 (40)	0.020
- Retinal Detachment, *n* (%)	3 (13.6)	3 (25.0)	0 (0)	0.960
Vision Loss, *n* (%)	19 (86.4)	12 (100)	7 (70)	0.046
Secondary Syphilis, *n* (%)	9 (40.9)	6 (50)	3 (30)	0.115
Latent Syphilis, *n* (%)	13 (59.0)	6 (50)	7 (70)	0.175
Concurrent Neurosyphilis, *n* (%)	6 (27.3)	6 (50)	0 (0)	**0.004**
LP (Lumbar Puncture) Performed, *n* (%)	16 (73)	9 (75)	7 (70)	0.789
CSF FTA-ABS Positivity, *n* (%)	5/10 (50.0)	5/6 (83.3)	0/4 (0)	**0.010**
CSF VDRL Positivity, *n* (%)	3/11 (27.3)	3/5 (60.0)	0/6 (0)	**0.020**
Blood Leukocyte Count/mm^3^ (*n*)	7414	6838	8105	0.330
Blood Neutrophil Count/mm^3^ (*n*)	4557	4282	4887	0.572
Blood Lymphocyte Count/mm^3^ (*n*)	2059	1800	2207	0.323
Systemic Steroid Use (*n*)	11	8	3	0.094

Note: Data are presented as number (percentage) unless otherwise stated. For CSF analyses, values represent the number of positive cases over the number of tested patients. Bold values indicate statistically significant results (*p* < 0.05).

**Table 2:** Sites of syphilis eye involvement in individuals living with and without HIV.

Eye Involvement	All Patients (*n* = 22) *n* (%)	PLWH (*n* = 12) *n* (%)	HIV-Negative (*n* = 10) *n* (%)	*p*
Type of uveitis involvement				
Anterior	3 (13.6)	1 (8.3)	2 (20.0)	0.550
Intermediate-Posterior	6 (27.3)	3 (25.0)	3 (30.0)	1.000
Pan	11 (50.0)	8 (66.7)	3 (30.0)	0.660

Note: Data are presented as number (percentage).

## Data Availability

Anonymized data supporting the findings of this study are available from the corresponding author upon reasonable request, subject to ethical approval and data protection regulations.

## Abbreviations

AIDS	Acquired Immunodeficiency Syndrome
ART	Antiretroviral Therapy
CDC	Centers for Disease Control and Prevention
CI	Confidence Interval
CNS	Central Nervous System
CSF	Cerebrospinal Fluid
FTA-ABS	Fluorescent Treponemal Antibody Absorption Test
HIV	Human Immunodeficiency Virus
IM	Intramuscular
IV	Intravenous
LP	Lumbar Puncture
MMWR	Morbidity and Mortality Weekly Report
MSM	Men Who Have Sex with Men
NHS	National Health Service
OR	Odds Ratio
PLWH	People Living with HIV
PrEP	Pre-Exposure Prophylaxis
SPSS	Statistical Package for the Social Sciences
TPHA	*Treponema pallidum* Hemagglutination Assay
VDRL	Venereal Disease Research Laboratory

## References

[1] Bennett J.E., Dolin R., Blaser M.J. (2019). Mandell, Douglas, and Bennett’s Principles and Practice of Infectious Diseases.

[2] World Health Organization (2023). Global Progress Report on HIV, Viral Hepatitis and Sexually Transmitted Infections, 2023: Accountability for the Global Health Sector Strategies 2022–2030.

[3] Republic of Türkiye Ministry of Health, Halk Sağlığı Genel Müdürlüğü (2023). Sifiliz Enfeksiyonu İstatistikleri, Türkiye, 2006–2023.

[4] Fonollosa A., Giralt J., Pelegrín L., Sánchez-Dalmau B., Segura A., García-Arumí J., Adan A. (2009). Ocular syphilis—Back again: Understanding recent increases in the incidence of ocular syphilitic disease. Ocul. Immunol. Inflamm..

[5] Sullivan P.S., Sanchez T.H., Zlotorzynska M., Chandler C.J., Sineath R., Kahle E., Tregear S. (2020). National trends in HIV pre-exposure prophylaxis awareness, willingness and use among United States men who have sex with men recruited online, 2013 through 2017. J. Int. AIDS Soc..

[6] Montaño M.A., Dombrowski J.C., Dasgupta S., Golden M.R., Manhart L.E., Barbee L.A., Duerr A., Khosropour C.M. (2019). Differences in sexually transmitted infection risk comparing pre-exposure prophylaxis users and propensity score-matched historical controls in a clinic setting. AIDS.

[7] Hamill M.M., Ghanem K.G., Tuddenham S. (2024). State-of-the-art review: Neurosyphilis. Clin. Infect. Dis..

[8] Tsan G.L., Claiborne R.T. (2021). Ocular syphilis. Clin. Exp. Optom..

[9] Ghanem K.G., Ram S., Rice P.A. (2020). The modern epidemic of syphilis. N. Engl. J. Med..

[10] Vadboncoeur J., Labbé A.C., Fortin C., Rabia Y., Aubin M.-J., Jaworsky L., Serhir B. (2020). Ocular syphilis: Case series (2000–2015) from two tertiary care centers in Montreal, Canada. Can. J. Ophthalmol..

[11] Oliver S.E., Aubin M., Atwell L., Matthias J., Cope A., Mobley V., Goode A., Minnerly S., Stoltey J., Bauer H.M. (2016). Ocular syphilis—Eight jurisdictions, United States, 2014–2015. MMWR Morb. Mortal. Wkly. Rep..

[12] Centers for Disease Control and Prevention (2015). Sexually Transmitted Diseases Treatment Guidelines, 2015.

[13] French P., Gomberg M., Janier M., Schmidt B., Vader P.v.V., Young H. (2009). IUSTI: 2008 European guidelines on the management of syphilis. Int. J. STD AIDS.

[14] Marra C.M., Maxwell C.L., Smith S.L., Lukehart S.A., Rompalo A.M., Eaton M., Stoner B.P., Augenbraun M., Barker D.E., Corbett J.J. (2004). Cerebrospinal fluid abnormalities in patients with syphilis: Association with clinical and laboratory features. J. Infect. Dis..

[15] Chow F. (2021). Neurosyphilis. Continuum.

[16] Oliver G.F., Stathis R.M., Furtado J.M., E Arantes T., McCluskey P.J., Matthews J.M., Smith J.R., International Ocular Syphilis Study Group (2019). Current ophthalmology practice patterns for syphilitic uveitis. Br. J. Ophthalmol..

[17] Mathew R.G., Goh B.T., Westcott M.C. (2014). British Ocular Syphilis Study (BOSS): A two-year national surveillance study of intraocular inflammation secondary to ocular syphilis. Investig. Opthalmology Vis. Sci..

[18] Furtado J.M., Arantes T.E., Nascimento H., Vasconcelos-Santos D.V., Nogueira N., Queiroz R.d.P., Brandão L.P., Bastos T., Martinelli R., Santana R.C. (2018). Clinical manifestations and ophthalmic outcomes of ocular syphilis at a time of re-emergence of the systemic infection. Sci. Rep..

[19] Fonollosa A., Martinez-Indart L., Artaraz J., Vasconcelos-Santos D.V., Nogueira N., Queiroz R.d.P., Brandão L.P., Bastos T., Martinelli R., Santana R.C. (2016). Clinical manifestations and outcomes of syphilis-associated uveitis in Northern Spain. Ocul. Immunol. Inflamm..

[20] Wells J., Wood C., Sukthankar A., Jones N.P. (2018). Ocular syphilis: The re-establishment of an old disease. Eye.

[21] Amaratunge B.C., Camuglia J.E., Hall A.J. (2010). Syphilitic uveitis: A review of clinical manifestations and treatment outcomes in HIV-positive and HIV-negative patients. Clin. Exp. Ophthalmol..

[22] Tran T.H., Cassoux N., Bodaghi B., Fardeau C., Caumes E., Lehoang P. (2005). Syphilitic uveitis in patients infected with human immunodeficiency virus. Graefes Arch. Clin. Exp. Ophthalmol..

[23] Shalaby I.A., Dunn J.P., Semba R.D., Jabs D.A. (1997). Syphilitic uveitis in human immunodeficiency virus–infected patients. Arch. Ophthalmol..

[24] Li J.Z., Tucker J.D., Lobo A.M., Marra C.M., Davis B.T., Papaliodis G.N., Felsenstein D., Durand M.L., Yawetz S., Robbins G.K. (2010). Ocular syphilis among HIV-infected individuals. Clin. Infect. Dis..

[25] He H., Wang M., Zaller N., Wang J., Song D., Qu Y., Sui X., Dong Z., Operario D., Zhang H. (2014). Prevalence of syphilis infection and associations with sexual risk behaviors among HIV-positive men who have sex with men in Shanghai, China. Int. J. STD AIDS.

[26] Musher D.M., Hamill R.J., Baughn R.E. (1990). Effect of human immunodeficiency virus infection on the course of syphilis and on the response to treatment. Ann. Intern. Med..

[27] Lee S.Y., Cheng V., Rodger D., Rao N. (2015). Clinical and laboratory characteristics of ocular syphilis: A new face in the era of HIV co-infection. J. Ophthalmic Inflamm. Infect..

[28] Gökengin D. (2022). HIV pre-exposure prophylaxis in Central and Eastern Europe—Gains and challenges in an ever—Changing world. Infect. Dis. Clin. Microbiol..

[29] Berg R.C., Lemke R., Ross M.W. (2017). Sociopolitical and cultural correlates of internalized homonegativity in gay and bisexual men: Findings from a global study. Int. J. Sex. Health.

[30] Sönmez İ., Berg R.C., Yazıcılaroğlu S.S., Thurlby N., Schmidt A.J. (2022). Comparison of the influence of internalized homonegativity on sexual risk behavior of men who have sex with men in Spain and Turkey. Int. J. Sex. Health.

